# Paraoxonase 3 gene polymorphisms are associated with occupational noise-induced deafness: A matched case-control study from China

**DOI:** 10.1371/journal.pone.0240615

**Published:** 2020-10-15

**Authors:** Huaping Zhou, Jinpeng Zhou, Hui Li, Changye Hui, Jing Bi

**Affiliations:** 1 Department of Occupational Health Surveillance, Shenzhen Prevention and Treatment Center for Occupational Diseases, Shenzhen, Guangdong Province, China; 2 Department of Occupational Disease Diagnosis, Shenzhen Prevention and Treatment Center for Occupational Diseases, Shenzhen, Guangdong Province, China; 3 Department of Occupational Disease, Shenzhen Prevention and Treatment Center for Occupational Diseases, Shenzhen, Guangdong Province, China; 4 Department of Pathology and Toxicology, Shenzhen Prevention and Treatment Center for Occupational Diseases, Shenzhen, Guangdong Province, China; University of Iowa, UNITED STATES

## Abstract

Chronic exposure to noise is a detrimental environmental factor that can contribute to occupational noise-induced deafness (ONID) in industrial workers. ONID is caused by both environmental and genetic factors, and negatively impacts workers and manufacturing industries in China. Polymorphisms in the paraoxonase 2 gene (*PON2*) is associated with noise-induced hearing loss, and *PON3* expression may modulate oxidative stress in cells and tissues by reducing the levels of reactive oxygen species, which are prominent in ONID. We conducted a matched case-control study to investigate whether *PON3* polymorphisms and activity were associated with susceptibility to ONID. We genotyped *PON3* single nucleotide polymorphisms (SNPs) using Sanger sequencing and measured the plasma *PON3* activity using enzyme-linked immunosorbent assay. Conditional logistic regression models were fitted to evaluate the potential risk factors of ONID. A total of 300 subjects were included (n = 150 ONID and n = 150 control cases) from October 2017 to October 2019. We identified two types of genotypes for the *PON3* SNPs. The independent risk factors for ONID were genotype CT and allele C with Odd’s ratio (OR) = 2.12 (95% confidence interval [CI]: 1.18–3.84) and OR = 1.68 (95% CI: 1.06–2.66) for SNP rs11767787; AG and allele A with OR = 2.09 (95% CI: 1.25–3.47) and OR = 1.87 (95% CI: 1.19–2.93) for SNP rs13226149; and CT and allele T with OR = 2.59 (95% CI: 1.44–4.67) and OR = 1.95 (95% CI: 1.22–3.14) for SNP rs17882539, respectively. Furthermore, the plasma *PON3* level (> 1504 U/L) was observed to be a protective factor associated with the lowest level of ONID (less than 991 U/L) after adjusting for confounding factors (OR = 0.27, 95% CI: 0.13–0.54). In conclusion, the *PON3* polymorphisms rs11767787, rs13226149, and rs17882539 and plasma *PON3* activity are associated with susceptibility to ONID in the Chinese population.

## Introduction

Noise is the most important environmental factor that may be detrimental to health [1, 2], especially for hearing loss [3]. Occupational noise is the most frequent occupational hazard, and the rate of disability-adjusted life years attributed to this form of noise has steadily increased worldwide from 1990 (61.11 /100 thousand) to 2017 (78.21/100 thousand) [4]. Noise-induced hearing loss (NIHL) is a sensorineural hearing deficit that begins with chronic exposure to the higher frequencies (3 to 6 kHz) and is the primary occupational disease predominantly found among industrial workers [1].

NIHL is a complex disease caused by the interaction between environmental factors and susceptibility genes [5]. The development of NIHL is mainly due to the duration of exposure, and the intensity and frequency of the noise, which results in cochlear epithelium damage. When industrial workers are exposed to excessive noise [8h ≥ 85 dB(A)] in the workplace, the cochlea consumes a lot of energy and then releases a large number of free radicals (reactive oxygen species and reactive nitrogen) locally [6]. As the antioxidant system is unable to neutralize these free radicals, the cochlear sensorial epithelium becomes damaged [7]. Consequently, genes involved in the regulation of reactive oxygen species, such as superoxide dismutase, glutathione S-transferase, and catalase, may affect the vulnerability of the cochlea to NIHL [8, 9].

Paraoxonases (*PONs*) are an aromatic esterase family that hydrolyze phosphate bond and degrade organophosphate compounds, aromatic carboxylates, and carbamates. The *PON* gene family of enzymes, which consists of *PON1*, *PON2*, and *PON3*, share approximately 60% and 70% identity at the amino acid, and nucleotide levels, respectively, in humans. *PONs* are acute phase proteins capable of degrading lipid peroxides, share considerable sequence identity, and span ~150 kb in tandem on the long arm of the human chromosome, 7q22.3−q22.1 [10, 11]. *PON* exerts antioxidant activity and protects against diseases such as hypertension, atherosclerosis, Alzheimer’s dementia, and Parkinson’s disease [3, 12–14]. Polymorphisms in the *PON2* gene are associated with susceptibility to NIHL in Chinese industrial workers [15, 16], and may have an interaction enhancement effect with noise exposure level and other factors [17].

*PON3* is the last member of the *PON* family of proteins to be described and is the least characterized. *PON3* is a 40-kDa glycoprotein that is synthesized in the liver in a calcium-dependent manner. It has limited aromatic esterase activity and retains lipo-lactonases and N-acyl-homoserine lactone activities but cannot hydrolyze organophosphate [18]. Like *PON1*, *PON3* is tightly bound to high-density lipoproteins in circulation, which enhance its antiatherosclerotic properties [19]. *PON3* has a higher catalytic activity for statin lactones than *PON1* [8, 20]. *PON3* also appears to modulate oxidative stress, similar to *PON1* and *PON2*, in cells and tissues by reducing reactive oxygen species. Previous studies have indicated that *PON3* is associated with coronary artery disease, atherosclerosis, chronic liver disease, and obesity [21–23]. Liu recommended that clinicians and patients be aware of the risk of atorvastatin-associated tinnitus and permanent hearing loss based on a case report [24]. *PON3* is the only enzyme that catalyzes the hydrolysis of a statin lactone ring, and a tightly linked group of *PON3* polymorphisms (rs11767787, rs13226149, and rs17882539) are thought to be associated with changes in the atorvastatin δ-lactone hydrolysis [25]. Thus, we hypothesized that genetic variation of the *PON3* gene and *PON3* activity may play an active role in increasing the susceptibility to NIHL.

Occupational noise-induced deafness (ONID) is the most serious level of NIHL and is regarded as an important element that influences the quality of social, familial, and professional life [17]. Shenzhen, located in the south of China with a 100% urbanization rate, is an international comprehensive transportation hub and had a permanent population of 1302.66 (10 000 persons) in 2018 [26]. There are more than 8000 industrial enterprises with noise hazards in Shenzhen, and approximately190 thousand workers were at risk of occupational noise in 2018. The ONID caused by long-term occupational exposure to noise has been the first occupational disease in Shenzhen for many years.

In light of the evidence that the allele frequencies for the known *PON2* polymorphisms are associated with NIHL and starting from the hypothesis that variation in the *PON3* gene may play an important role in human ONID development. We aimed to identify the associations between *PON3* polymorphisms and *PON3* activity and susceptibility to ONID after long-term occupational exposure to noise. The outcomes obtained from the current investigation are expected to help screen individuals sensitive to noise. We hope our findings help reduce the occurrence of ONID and improve working conditions in Shenzhen, China.

## Materials and methods

### Study population

We focused on ONID cases in Shenzhen industrial workers. We conducted a matched case-control study to investigate the potential relationship between ONID and *PON3* polymorphisms between October 1, 2017 and October 31, 2019 in Shenzhen. All participants were Han Chinese employees who were recruited from manufacturing units and industries that were noise prone such as mechanical tools, domestic appliance production and transportation, building and steel construction, respectively. All these workers were commonly exposed to a steady stream of noise [LEX, 8h ≥ 85 dB(A)] with at least three years of exposure to work-related noise. Workers with hypertension, diabetes, hyperlipidemia, cardiovascular events, history of head injury, family history of deafness, ear infection, ontological disease, drug-induced deafness, and those with other obvious causes of hearing loss (such as high temperature, explosives, heavy metals, and organic solvents) were excluded from the study. ONID cases were newly diagnosed at the Shenzhen Prevention and Treatment Center for Occupational Diseases according to the diagnosis of occupational noise-induced deafness (GBZ 49–2014) [27]. The gender, age (±2 years) and exposure time (±1 year) matched controls, consisted of healthy employees from the same company who were seeking health checkups from the Shenzhen Prevention and Treatment Center for Occupational Diseases at the same time.

### Covariate assessment

A standardized, structured questionnaire was used to collect the potential occupational and risk factors for hearing loss of participants. All of them were interviewed face-to-face by trained medical workers. The questionnaire covered four types of information, including sociodemographic characteristics (age, gender, height, weight and hereditary factors), diseases (previous and present medical conditions, pharmaceutical preparations and history of head injury), participants lifestyles (smoking and drinking status), and occupation-related factors (type of work, noise exposure time for work, and leisure). The self-reported diseases were verified by specialists at the Shenzhen Prevention and Treatment Center for Occupational Diseases according to recognized international standards. The subjective effects such as tinnitus, vertigo and sensation of fullness in the ears were investigated. Smoking and drinking statuses were defined by subjects who had a minimum of one cigarette per day for at least one year and drank 50 g of wine or a bottle of beer per day for at least one year, respectively [15].

### Noise intensity

We used a noise statistical analyzer (Quest SoundPro, Quest Inc., USA) to measure the intensity of noise in the workplace during the working time. As recommended by the Occupational Health Standard of the People’s Republic of China: Measurement of physical agents in the workplace—part 8: Noise [GBZ/T 189.8–2007] [28], the noise statistical analyzer was set at the ear height (about 1.5 m in the standing position and 1.1 m at the seat), with a microphone in the direction of the noise source. Each location was measured three times, and the noise exposure level for individuals was averaged. If the participants left the original noise-exposed environment, the data of occupational risk factors in previous workplaces were supported by a previous employer.

### Hearing threshold

The pure-tone thresholds for each ear of all the participants were obtained using a GIS-61 audiometer (Grason-Stadler, Eden Prairie, MN) with a TDH-50P headphone (Grason-Stadler, Eden Prairie, MN) at six audiometric frequencies (0.5, 1, 2, 3, 4, and 6 kHz). Audiometry was conducted in a soundproof room by a trained technician and the background sound level was less than 30 dB(A). Hearing thresholds were adjusted by age and sex according to the Occupational Health Standard of the People’s Republic of China: Acoustics-statistical distribution of hearing thresholds as a function of age [GB/T 7582–2004] and the diagnosis of occupational noise-induced deafness (GBZ 49–2014) [27]. The hearing thresholds were classified into three types: mild [hearing threshold: 26–40 dB(A)], moderate [hearing threshold: 41–55 dB(A)] and severe [hearing threshold: ≥ 56 dB(A)].

### SNP genotyping

According to Stephan et al., we selected the *PON3* single nucleotide polymorphisms (SNPs) rs11767787, rs13226149, and rs17882539 to be genotyped by Sanger sequencing [25]. All ONID cases and controls donated 5 mL of fasting peripheral venous blood samples using EDTA-K2 anticoagulation tubes in the morning. Ezup column-based genomic blood DNA extraction kit (Sangon Biotech, China) was used to extract genomic DNA according to the manufacturer’s protocols [29]. A UV-Vis spectrophotometer (SMA4000; Merinton, China) and 1% agarose gel electrophoresis (AGE) (FR-980A; Shanghai Furi Technology Co., LTD, China) were used to test the quality of DNA. The primers (Table 1) for the three *PON3* SNPs (rs11767787, rs13226149, and rs17882539) were designed using the Primer Premier 5 software. Polymerase chain reaction (PCR) amplification (Verity 96well; Applied Biosystems, USA) was performed using the same conditions for three *PON3* SNPs: 5 min at 95°C, followed by 38 cycles of denaturation for 30 s at 94°C, annealing for 30 s at 58°C, and extension for 60 s at 72°C, with a final extension at 72°C for 10 min. The fragment of PCR products was tested using 1% agarose gel electrophoresis (AGE). The then *PON3* SNPs (rs11767787, rs13226149, and rs17882539) were then genotyped using a 3730XL sequencer (Applied Biosystems, USA). As a quality control, 5% of the randomly selected samples were genotyped as blinded duplicates, and the results were 100% concordant.

**Table 1:** Primer sequence and characteristics for *PON3* SNPs.

SNP	Sequence (5'-3')	Genomic position	Region	L(bp)	Base Change
rs11767787	Forward: GAGTTCGGGCAAAGTAGCAC	3935	Promoter	546	A/G
	Reverse: CTCTGCTTGCACTCTTTTAACATTA				
rs13226149	Forward: CCAAGCAGAATGTTGAGGGC	5088	Exonic	705	C/T
	Reverse: GTAGGATTTCGCGGGTGTTA				
rs17882539	Forward: TGACCTACAGCAAGCCACA	4280	Promoter	877	C/T
	Reverse: AACACCCGCGAAATCCTA				

SNP, single nucleotide polymorphism; L, length of amplicons.

### Measurement of plasma *PON3* activity

Peripheral venous blood samples were centrifuged at 2500 rpm for 20 min, and the supernatant was collected to determine human plasma *PON3* activity using an enzyme-linked immunosorbent assay Kit (96T; Sangon Biotech, China) according to the manufacturer’s protocols. Absorbance readings were measured at 450 nm using a microplate reader (RT-6100; Rayto, USA) with a blank hole as a reference. The results were then converted into concentrations by comparison with standard curve values. All measurements were performed in duplicate and expressed as U/L (1U = 1 mol of paraoxon hydrolyzed per minute). The correlation coefficient between the samples and the expected concentration was above 0.99, and the coefficient of variation within batches and between batches was less than 10% and 15%, respectively.

### Sample size

We used two correlated (paired) proportions to calculate the sample size of this matched case-control study using the PASS 15 software. For each case patient, a matching sample of one control was also obtained. The probability of exposure (CT+TT for rs11767787; AA+AG for rs13226149 and CT+TT for rs17882539) among the control population was 0.20, and the correlation coefficient for exposure between ONID case and control population is 0.25. The power was 90% and the odds ratio (OR) was 2.00 with a = 0.05. The calculated sample size was 130 for each group; we amplified 15% of the sample size considering the refusal, and obtained a sample size of 150 ONID patients and 150 controls.

### Ethical approval

The analysis presented in this manuscript is based on data and specimens in accordance with the guidelines of the Declaration of Helsinki, and all identified individual information was anonymized. This project was approved by the Ethics Committee of Shenzhen Prevention and Treatment Center for Occupational Diseases. All participants in this study signed an informed consent form before participating.

### Statistical analysis

The Shapiro–Wilk test was used to check the normality of distribution of continuous variables, and mean ± standard deviation (SD) or median (interquartile range, IQR) were presented according to whether they were normally distributed. The Paired-samples *t*-test or Wilcoxon signed ranks test was used to detect significant differences between the ONID and control groups for continuous variables. Categorical variables were described as frequencies and percentages. The McNemar-Bowker test was used to compare the differences between the ONID and control groups. The chi-square test was used to check for deviations from the Hardy-Weinberg equilibrium (HWE) for each SNP in the control group. Conditional logistic regression models taking the matching into account were fitted to evaluate the potential relationship between *PON3* polymorphisms and the risk of ONID. The multivariate analyses were adjusted for covariates (age, smoking, drinking, exposure time, and noise exposure level) with crude and adjusted ORs with their respective 95% confidence intervals (95% CIs). Furthermore, the association of genotypes with ONID was also evaluated by assuming allele models. ORs and their 95% CIs were also calculated by including the exposure plasma *PNO3* activity, which was categorized into quintiles (less than P_25_, P_25_-P_50_, P_50_-P_75_, greater than P_75_), considering the lowest quintile as the reference. Statistical significance was set at *p*<0.05. All analyses were performed with R software, version 3.5.1.

## Results

### Characteristics of the participants

A total of 300 subjects were included (n = 150 ONID and n = 150 control cases) from October 2017 to October 2019. All the participants were mainly from Pingshan, Baoan, and Longgang districts, where the manufacturing industry is clustered. Of the 150 ONID cases, 139 (92.67%) and 11 (7.33%) were mild and moderate, respectively. There were no severe ONID cases in this study. The mean age was 44.37±7.55 years (range, 23–61 years) and 94% were male. The median exposure time was 11.42 (7.75) years and the intensity of noise was from 85 to 113 dB(A), with a mean noise exposure level of 93.11±5.99 dB(A). Furthermore, 34.67% and 41.67% of the study population were smoking and drinking, respectively, and 87.33% of them worked in manufacturing. There were no statistical differences between the ONID and control groups regarding age, gender, smoking, drinking, exposure time, and noise exposure level (Table 2).

**Table 2:** Demographics of ONID cases and control population.

Variables	Total (n = 300)	ONID group (n = 150)	Control group(n = 150)	*P* value
Age, years	44.37±7.55	44.34±7.53	44.41±7.59	0.565
Gender, n (%)				1.000
Male	282(94)	141(94)	141(94)	
Female	18(6)	9(6)	9(6)	
BMI (kg/m^2^)	21.59±1.20	21.67±1.16	21.51±1.23	0.246
Smoking, n (%)				0.659
No	196(65.33)	96(64)	100(66.67)	
Yes	104(34.67)	54(36)	50(33.33)	
Drinking, n (%)				0.569
No	175(58.33)	90(60)	85(56.67)	
Yes	125(41.67)	60(40)	65(43.33)	
Type of work, n (%)				1.000
Manufacturing	262(87.33)	131(87.33)	131(87.33)	
Construction	8(2.67)	4(2.67)	4(2.67)	
Transportation	18(6)	9(6)	9(6)	
Others	12(4)	6(4)	6(4)	
Exposure time, years, M (IQR)	11.42(7.75)	11.29(7.69)	11.54(8)	0.363
Noise Exposure Level, Db (A)	93.11±5.99	93.09±5.70	93.12±6.30	0.965

ONID, occupational noise-induced deafness; M, median; IQR, interquartile range.

### Hearing thresholds

**Fig 1:**
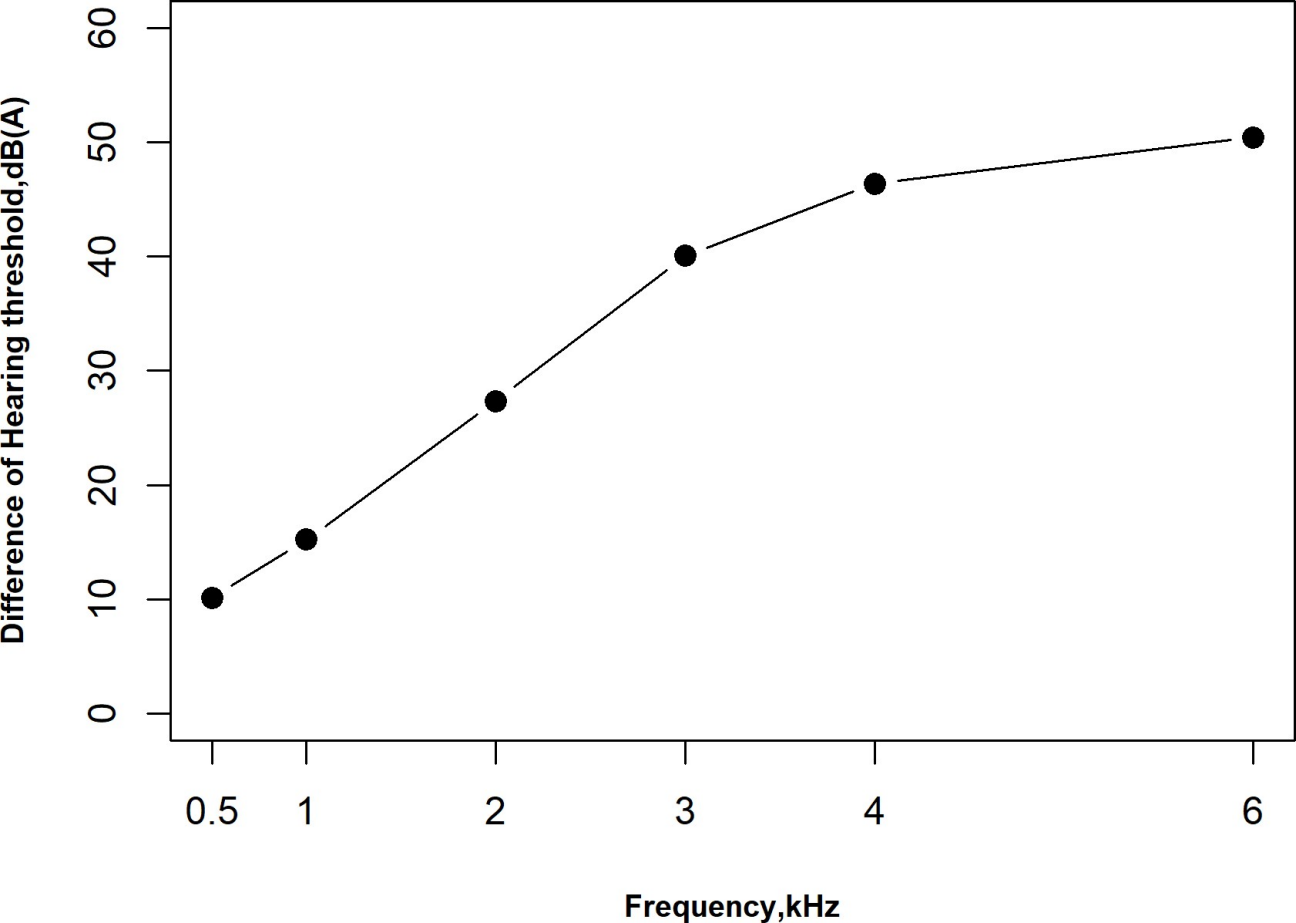
Mean differences in hearing thresholds between ONID and control groups at all frequencies. The differences of hearing thresholds between ONID and control group at frequency 3.0, 4.0 and 6.0 kHz were observed to be stable. Data are expressed as mean difference.

The distribution of mean hearing thresholds at frequencies from 0.5 to 6.0 kHz are shown in Table 3. The mean hearing thresholds increased as the frequency increased in both groups, and hearing thresholds were significantly higher in the ONID group than in the control group at all frequencies. The difference in hearing thresholds between the ONID and control groups also increased as the frequency increased (Fig 1).

**Table 3:** The distribution of hearing thresholds at different frequencies in the ONID and control groups.

Groups	0.5 kHz	1.0 kHz	2.0 kHz	3.0 kHz	4.0 kHz	6.0 kHz
ONID group (n = 150)	24.20±7.97	30.82±9.11	42.93±12.80	56.60±13.33	63.57±13.74	67.97±16.41
Control group (n = 150)	14.07±5.78	15.60±5.05	15.57±5.26	16.53±5.37	17.17±4.86	17.53±4.63
*t*	12.229	17.450	23.549	34.412	38.774	37.080
*P* value	<0.001	<0.001	<0.001	<0.001	<0.001	<0.001

ONID, occupational noise-induced deafness.

### Fragment of PCR amplification

**Fig 2:**
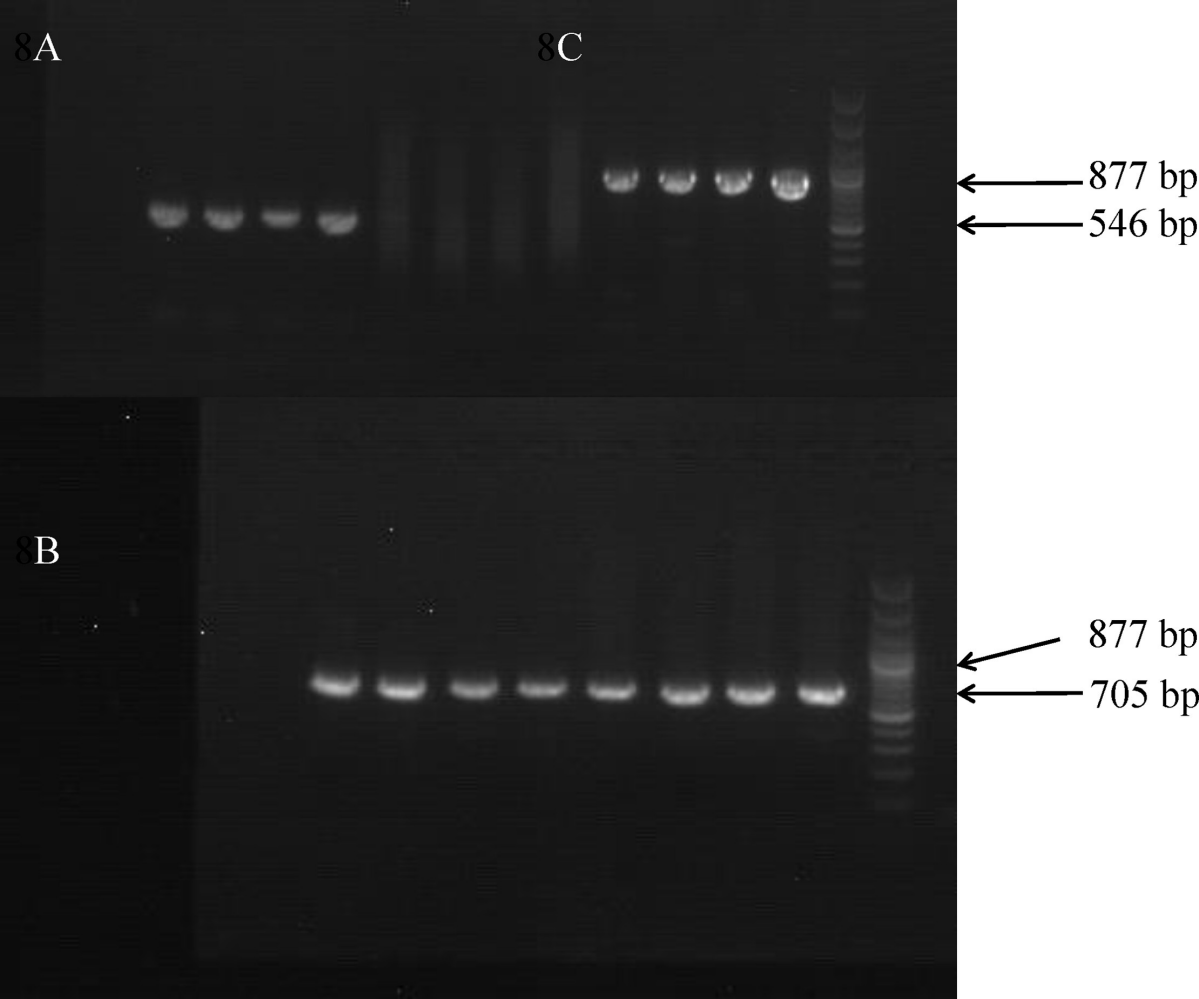
The electrophoresis of *PON3* PCR amplicons for gene. (A) rs11767787, (B) rs13226149 (C) rs17882539.

The fragments of PCR amplification for the *PON3* gene SNPs are shown in Fig 2. The electrophoresis results showed that the extraction products of rs11767787 (546 bp), rs13226149 (705 bp), and rs17882539 (877 bp) obtained a single band with an appropriate position and clear background.

### Hardy-Weinberg equilibrium

The genotype information for the three SNPs of the *PON3* gene in the control population are displayed in Table 4. All of the observed genotype frequencies were in agreement with the HWE (*p* > 0.05).

**Table 4:** The HWE for *PON3* polymorphisms in control population.

SNPs	Genotypes	Observed	Theoretical	*P* value
rs11767787	TT	115	117.04	0.106
	CT	35	30.92	
	CC	0	2.04	
rs13226149	GG	118	119.71	0.144
	AG	32	28.59	
	AA	0	1.70	
rs17882539	CC	120	121.50	0.174
	CT	30	27	
	TT	0	1.5	

SNP, single nucleotide polymorphism.

### Association of ONID with *PON3* SNPs

Only two genotypes were detected for three *PON3* SNPs (rs11767787, rs13226149, and rs17882539) in our study population. The genotypes and alleles are shown in Table 5. There were significant differences between the ONID and control groups in the distributions of genotypes and alleles for both rs11767787, rs13226149 and rs17882539 (*p <* 0.05).

**Table 5:** Associations of ONID risk with genotype and allele distributions of *PON3* polymorphisms.

SNPs	Models	Total (n = 300)	ONID group	Control group (n = 150)	*P* value	Crude OR	Adjusted OR*
			(n = 150)			(95% CI)	(95% CI)
rs11767787	Genotype				0.013		
	TT	211(70.33)	96(64)	115(76.67)		1	1
	CT	89(29.67)	54(36)	35(23.33)		2.12(1.19–3.77	2.12(1.18–3.84)
	Allele				0.013		
	T	511(85.17)	246(82)	265(88.33)		1	1
	C	89(14.83)	54(18)	35(11.67)		1.65(1.05–2.6)	1.68(1.06–2.66)
rs13226149	Genotype				0.003		
	GG	211(70.33)	93(62)	118(78.67)		1	1
	AG	89(29.67)	57(38)	32(21.33)		2.09(1.27–3.43)	2.09(1.25–3.47)
	Allele				0.003		
	G	511(85.17)	243(81)	268(89.33)		1	
	A	89(14.83)	57(19)	32(10.67)		1.86(1.19–2.91)	1.87(1.19–2.93)
rs17882539	Genotype				0.0009		
	CC	215(71.67)	95(63.33)	120(80)		1	1
	CT	85(28.33)	55(36.67)	30(20)		2.56(1.44–4.57)	2.59(1.44–4.67)
	Allele				0.0009		
	C	515(85.83)	245(81.67)	270(90)		1	
	T	85(14.17)	55(18.33)	30(10)		1.97(1.23–3.16)	1.95(1.22–3.14)

The numbers in the parentheses are % in columns 3, 4, and 5.

SNP, single nucleotide polymorphism; ONID, occupational noise-induced deafness; OR, odds ratio; 95% CI, 95% confidence interval.

*Adjusted for age, smoking, drinking, BMI, exposure time and noise exposure level in the conditional logistic regression model.

For rs11767787, there was an enrichment of CT (36%) genotype and allele C (18%) in the ONID cases, and a higher proportion of TT (76.67%) in the control group than in the ONID group (*p* = 0.013). After adjusting for confounding factors of age, smoking, drinking, BMI, exposure time, and noise exposure level in the conditional logistic regression model, there was a significant association between genotype CT and ONID compared with TT (OR = 2.12, 95% CI: 1.18–3.84, *p* = 0.01). Meanwhile, allele C was found to be a risk factor for ONID after adjusting for the confounders mentioned above (OR = 1.68, 95% CI: 1.06–2.66, *p* = 0.03).

The AG genotypes and allele A of rs13226149 were significantly more predominant in the ONID cases than in the control group (*p <* 0.05). The multivariate analysis results showed that the risk factors for ONID were genotypes AG and allele A, with OR = 2.09 (95% CI: 1.25–3.47, *p* = 0.005) and OR = 1.87 (95% CI: 1.19–2.93, *p* = 0.007), respectively.

For rs17882539, ONID cases carrying the CT genotype or allele T were significantly higher than in the control population (*p* < 0.05). The multivariate analysis results after adjusting for age, smoking, drinking, BMI, exposure time, and noise exposure level showed that the risk factors for ONID were genotypes CT and allele T, with OR = 2.59 (95% CI: 1.44–4.67, *p* = 0.001) and OR = 1.95 (95% CI: 1.22–3.14, *p* = 0.006), respectively.

### Characteristics of plasma *PON3* activity

**Fig 3:**
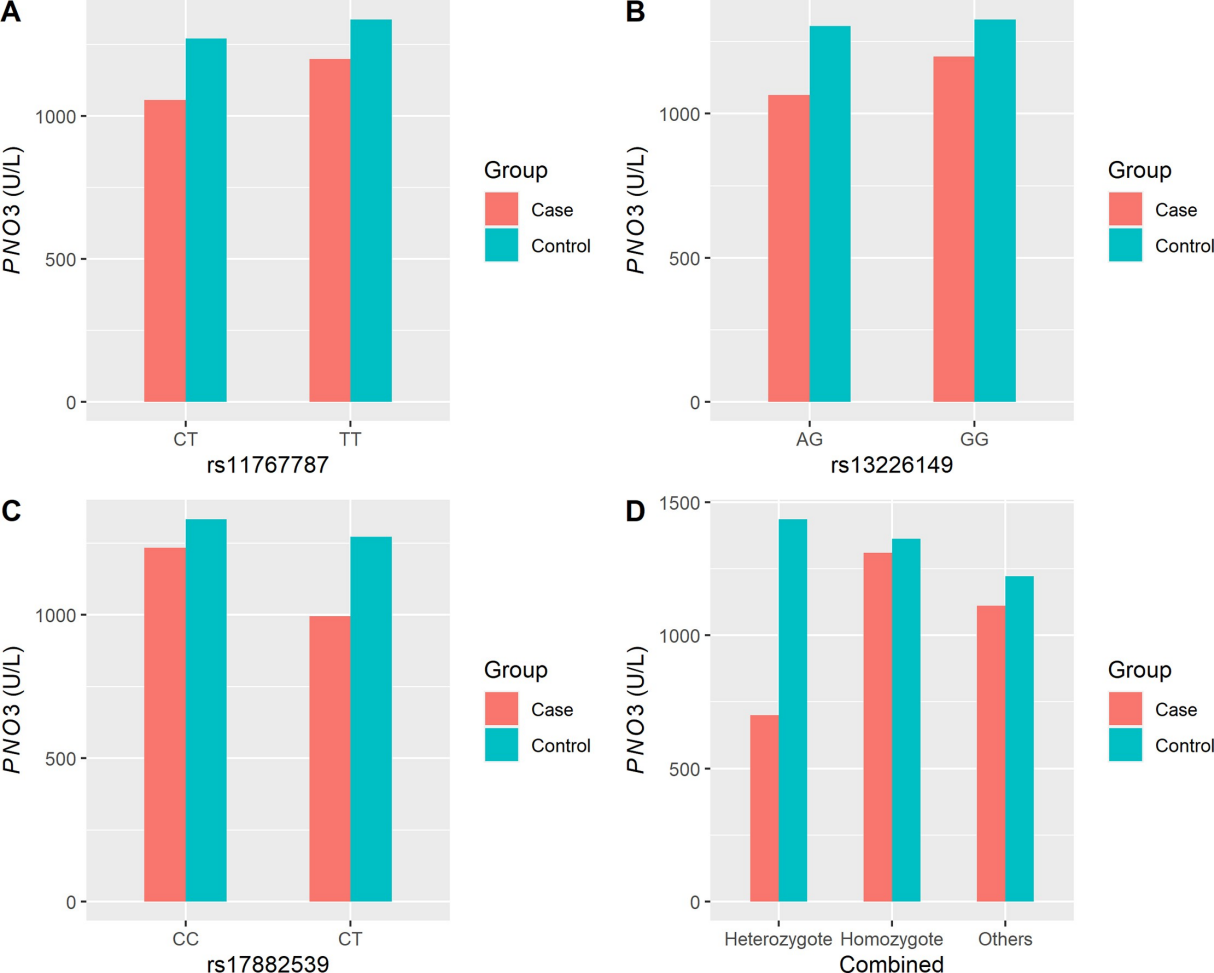
Distributions of plasma *PNO3* activity in the ONID and control groups for rs11767787, rs13226149 and rs17882539, and combined, respectively. Red bars represent control group and blue bars represent ONID cases. The genotypes are indicated below each bar chart. A showed *PON3* activity at rs11767787, B showed *PON3* activity at rs13226149, C showed *PON3* activity at rs17882539, and D showed *PON3* activity at three SNPs combined.

Plasma *PON3* activity in the ONID group was 1147.04±379.48 U/L, which was significantly lower than that of the control group (1322.24±374.64 U/L). We then we detected significant differences in the plasma *PON3* activity between the three *PON3* SNPs rs11767787, rs13226149, and rs17882539 between the ONID and control groups (*p* < 0.05), except for genotype CC of rs17882539 (Fig 3).

Furthermore, all participants were divided into homozygote, heterozygote, and other subgroups. Subjects caring for genotypes of TT, GG, and CC for rs11767787, rs13226149, and rs17882539, respectively, were divided into the homozygous group. Subjects caring genotypes of CT, AG, and CT for rs11767787, rs13226149, and rs17882539, respectively, were divided into the heterozygous group. All others were divided into the other sub-group. There were significant differences in the heterozygous genotype distributions between the ONID and control groups (*p* < 0.05); however, no significant differences were found in the homozygous and other subgroups between ONID and control population (Fig 3).

### Association of plasma *PON3* activity with ONID

**Fig 4:**
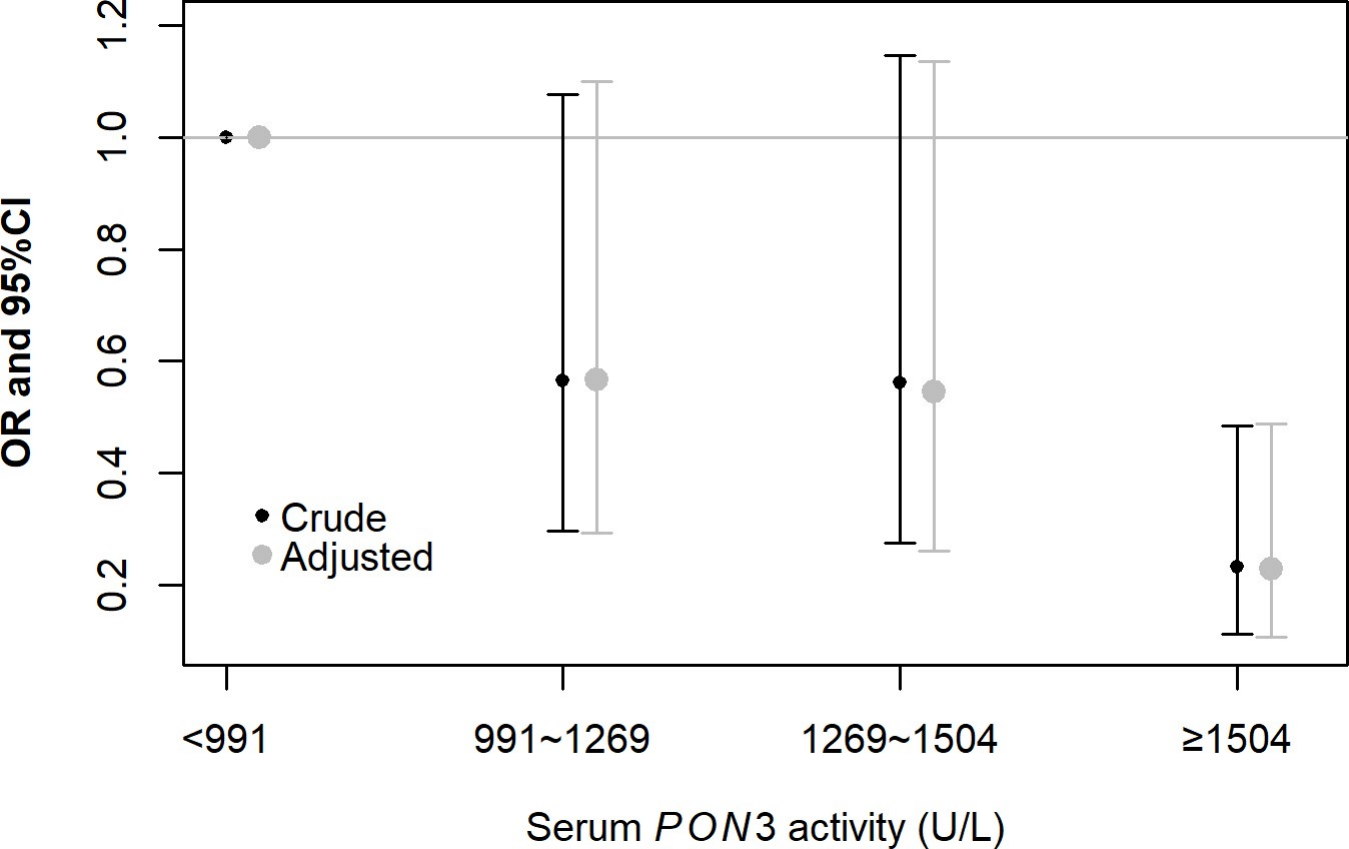
The risk of plasma *PON3* activity is associated with ONID in four groups. The exposure plasma *PNO3* activity was categorized into four groups (less than P_25_, P_25_-P_50_, P_50_-P_75_, greater than P_75_), considering the lowest group as the reference.

Plasma *PNO3* activity was categorized into four groups according to the IQR (Fig 4). In multivariate analysis adjusting for age, smoking, drinking, BMI, exposure time, and noise exposure levels in the conditional logistic regression model, the OR of ONID decreased 0.23-fold (95% CI: 0.11–0.49, *p* = 0.0001) in the highest quantile category (more than 1504 U/L) compared with the lowest quantile category (less than 991 U/L). However, we did not find statistically significant associations between the other two plasma *PNO3* activity categories and ONID in the multivariate analysis (*p* > 0.05).

## Discussion

To the best of our knowledge, this is the first study on the relationship between the *PON3* gene polymorphisms and susceptibility to ONID in industrial workers in China. In this matched case-control study, only two genotypes were isolated from peripheral venous blood samples for *PON3* gene SNPs rs11767787, rs13226149, and rs17882539. Significant differences between the ONID and control groups were observed in the distribution of genotypes and alleles for both rs11767787, rs13226149, and rs17882539 (*p <* 0.05). The independent risk factors for ONID were genotype CT and allele C, AG and allele A and CT and allele T for rs11767787, rs13226149, and rs17882539, respectively, irrespective of age, smoking, drinking, BMI, exposure time, and noise exposure level. Furthermore, the plasma *PON3* activity in the ONID group was significantly lower than that of the control group and the same as in different genotypes for three *PON3* SNPs (rs11767787, rs13226149, and rs17882539). Plasma *PON3* level (> 1504 U/L) was observed to be a protective factor associated with the lowest level of ONID (less than 991 U/L) adjusting for age, smoking, drinking, BMI, exposure time, and noise exposure level.

The development of ONID depends on factors concerning noise such as the duration of exposure and the intensity and frequency of the noise. The individual susceptibility to noise is hypothesized to correlate with hearing loss, including increasing age, male sex, smoking, and alcohol [30–33]. We conducted a matched case-control study to investigate the potential relationship between ONID and *PON3* polymorphisms in Shenzhen, and age, gender, and duration of exposure time were matched to the ONID cases to exclude the important confounders. Meanwhile, other confounders such as smoking, drinking, and noise exposure level were also balanced in our study. We found that the ONID group exhibited higher hearing thresholds at frequencies from 0.5 to 6.0 kHz in comparison with the control group. The differences in hearing thresholds between the ONID and control groups at frequency 3.0, 4.0, and 6.0 kHz were observed to be stable, consistent with the occurrence and development of ONID.

Individual susceptibility to noise is another important factor associated with inner ear vulnerability to noise after equivalent noise exposure. The etiology of ONID, which remains an unavoidable problem in industrial workers, involves an interaction between environmental and genetic factors. In fact, studies have shown that the concentrations of superoxide radicals in the cochlear fluid as well as in the stria vascularis increased after exposure to noise in animal models. Thus, genes involved in the regulation of reactive oxygen species, such as antioxidant activity *PONs* genes, may affect the vulnerability of the cochlea to ONID [16]. All *PON* genes are antioxidants and can hydrolyze a variety of substrates. There are several positive associations between the *PON2* gene and NIHL populations in Italy [34] and the Chinese population [17]. The association of *PON3* gene with ONID has, to our knowledge, not been investigated in a single comprehensive data set. Our data showed that the allelic variants for SNP rs11767787, rs13226149, and rs17882539 were rare (frequency *<* 1%) in this population. No homozygous (CC, AA, and TT) individuals for these mutations were identified. However, the results still found that the heterozygous alleles C, A, and T for SNP rs11767787, rs13226149, and rs17882539 were all associated with OIND, suggesting that individuals carrying the alleles C, A, and T may be more prone to ONID when exposed to occupational noise. The exact mechanisms involved remain unknown, however, warrant further investigation.

We also evaluated the effect of the variant on the metabolic activity of the *PON3* gene. *PON1* and *PON3* are found in the serum, whereas *PON2* is primarily an intracellular enzyme [35]. Our results suggest that low plasma *PON3* activity is a risk factor for OIND, especially for individuals with less than 991 U/L. Furthermore, we also found that plasma activities in the heterozygous ONID cases were significantly lower than in the control population for rs11767787, rs13226149 and rs17882539, and combined. Meanwhile, the levels of plasma *PON3* activity in the homozygous group were higher than in the heterozygous group in both ONID cases and the control population for the three SNPs. However, a slightly higher level of *PNO3* activity was found in the heterozygous subgroup compared the that of the homozygous subgroup for SNPs combined. This may be biased by the small sample size, only nine controls contained the heterozygote for the three SNPs together. Thus, further research is needed to illustrate the relationship between the genotype and *PNO3* activity. Our findings suggested that the *PON3* variants could strongly influence *PON3* activity, and that the overproduction heterozygous genotype may be capable of lowering the antioxidant activity. Serum *PON* activity is reduced in patients with Alzheimer’s disease [36] and systemic lupus erythematosus [37], which was consistent with our results.

There are some limitations to our study. First, the case-control study did not explain the causal relationship, whether the ONID was caused by *PON3* variants or vice versa. Second, the research subjects were all Chinese. Due to ethnic differences, more studies are needed to better understand the associations of *PON3* with the risk of developing ONID. Third, the sample size was small in our study; the homozygotes CC, AA, and TT for SNP rs11767787, rs13226149, and rs17882539 were not identified. A larger sample size and comprehensive resequencing may help to better understand the association analysis of the *PON3* gene with the risk of ONID in future work.

## Conclusions

In conclusion, we suggest that the *PON3* variants, rs11767787 C allele, rs13226149 A allele, and rs17882539 T allele and their associated plasma enzyme activities may increase the risk of developing ONID in Chinese industrial workers independent of age, smoking, drinking, BMI, exposure time, and noise exposure level. We also found that *PON3* variants strongly influenced the *PON3* activity and may be potential biomarkers for detecting ONID in noise-exposed workers.

## Supporting information

S1 File Information of each subject. Demographic characteristics and genotype result of each subject.
(CSV)

S2 File A completed STROBE checklist about case-control study. (DOCX)

S3 File (DOCX)

S4 File (DOCX)

S1 Raw images (PDF)

S1 Fig (TIF)

S2 Fig (JPG)
