# Supplementary material for: Paraoxonase 3 gene polymorphisms are associated with occupational noise-induced deafness: A matched case-control study from China
Source: PLoS One. 2020 Oct 15;15(10):e0240615. doi: 10.1371/journal.pone.0240615 (PMC7561195; doi:10.1371/journal.pone.0240615)
Supplement: S3 File — (DOCX) [file pone.0240615.s003.docx]

**Questionnaire**

**一﹑Sociodemographic characteristics**

1. Age：_______years
2. Height：_______m
3. Weight：_______Kg
4. Sex： □1=Male □2=Female
5. Race： □1=Han □2=Others
6. Type of work： □1= Manufacturing □2= Construction

□3= Transportation (Storage or postal service) □4=Others

1. Exposure time： __________years

二、**Lifestyle and related disease**

1. Smoking： □0=No □1=Yes
2. Drinking： □0=No □1=Yes
3. Diabetes： □0=No □1=Yes
4. Hypertension： □0=No □1=Yes
5. Hyperlipidemia： □0=No □1=Yes
6. Cardiovascular events： □0=No □1=Yes
7. History of head injury： □0=No □1=Yes
8. Family history of deafness： □0=No □1=Yes
9. Hearing loss： □0=No □1=Yes

**Auditor**  **Interviewer**

Name：____________ Name：____________

Date: _____y/_____m/____d Date: _____y/_____m/____d
