# Supplementary material for: Paraoxonase 3 gene polymorphisms are associated with occupational noise-induced deafness: A matched case-control study from China
Source: PLoS One. 2020 Oct 15;15(10):e0240615. doi: 10.1371/journal.pone.0240615 (PMC7561195; doi:10.1371/journal.pone.0240615)
Supplement: S4 File — (DOCX) [file pone.0240615.s004.docx]

**调查表**

**一﹑基本情况 (请在适合您的项目上打”√”)**

1. 年龄：_______岁
2. 身高：_______米
3. 体重：_______千克
4. 性别： □1=男 □2=女
5. 民族： □1=汉族 □2=其他民族
6. 职业： □1=制造业 □2=建筑业 □3=交通运输（仓储、邮政） □4=其他
7. 从业年限： __________年

**二﹑生活习惯及相关伴随疾病(请在适合您的项目上打”√”)**

1. 吸烟： □0=否 □1=是
2. 饮酒： □0=否 □1=是
3. 糖尿病： □0=否 □1=是
4. 高血压： □0=否 □1=是
5. 高血脂： □0=否 □1=是
6. 心血管事件： □0=否 □1=是
7. 头部损伤史： □0=否 □1=是
8. 家人是否有耳聋： □0=否 □1=是
9. 是否确诊过听力损失： □0=否 □1=是

**审核员**   **调查员**

姓名：____________ 姓名：____________

日期: ______年____月____日 日期: ______年____月____日
