# Supplementary material for: Paraoxonase 3 gene polymorphisms are associated with occupational noise-induced deafness: A matched case-control study from China
Source: PLoS One. 2020 Oct 15;15(10):e0240615. doi: 10.1371/journal.pone.0240615 (PMC7561195; doi:10.1371/journal.pone.0240615)
Supplement: S1 Raw images — (PDF) [file pone.0240615.s005.pdf]

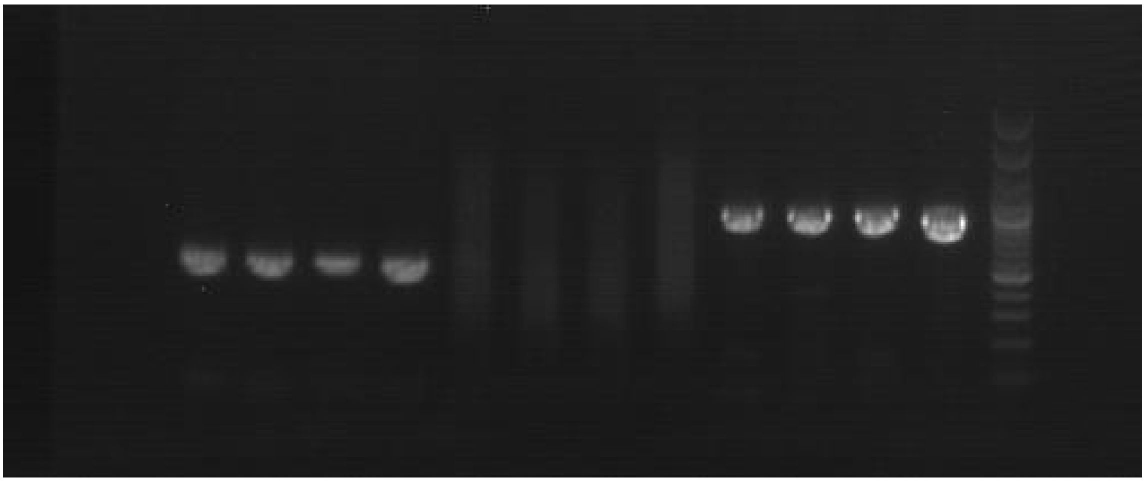

This is the raw gel image for Figure 2, the left plot is for rs11767787, and the right is for rs17882539.

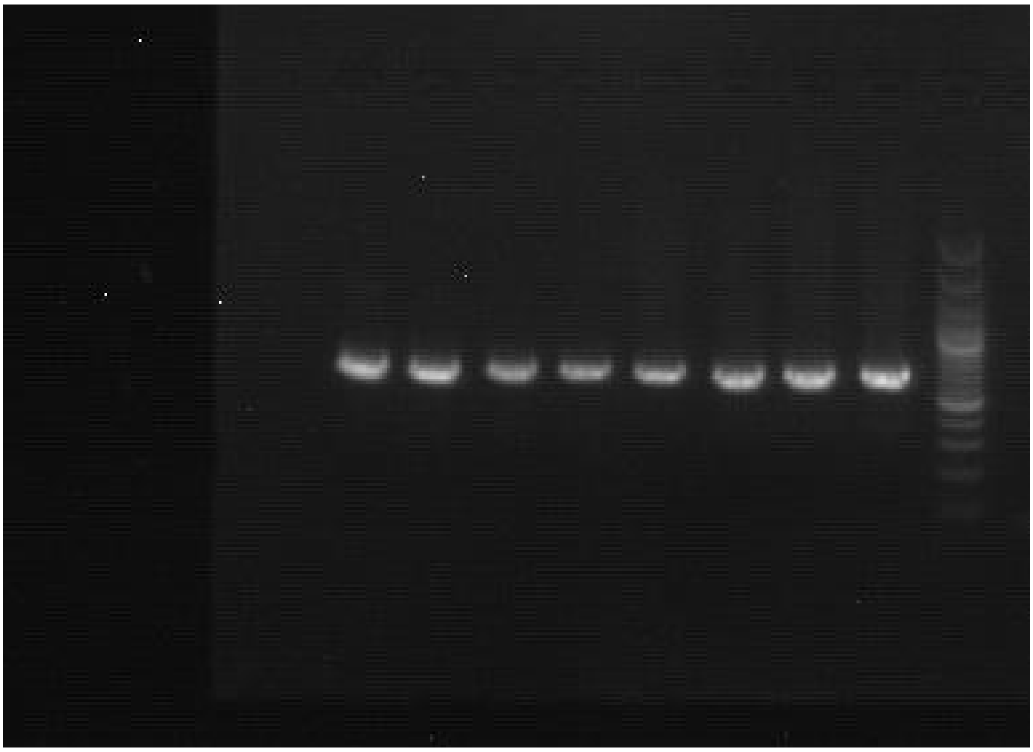

This is the raw gel image for Figure 2, the plot is for rs13226149.
