# Supplementary figures and images for: Paraoxonase 3 gene polymorphisms are associated with occupational noise-induced deafness: A matched case-control study from China
Source: PLoS One. 2020 Oct 15;15(10):e0240615. doi: 10.1371/journal.pone.0240615 (PMC7561195; doi:10.1371/journal.pone.0240615)

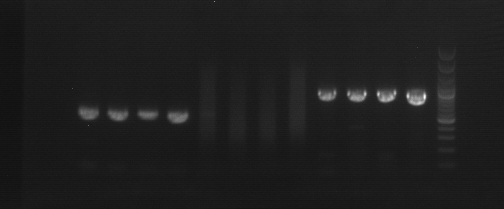

Supplement: S1 Fig — (TIF) [file pone.0240615.s006.tif]

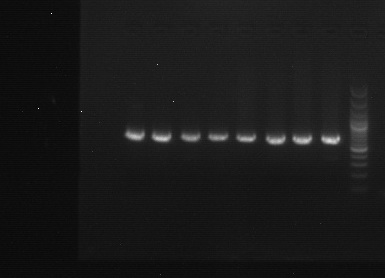

Supplement: S2 Fig — (JPG) [file pone.0240615.s007.jpg]
